# Potential-tuned selective electrosynthesis of azoxy-, azo- and amino-aromatics over a CoP nanosheet cathode

**DOI:** 10.1093/nsr/nwz146

**Published:** 2019-10-01

**Authors:** Xiaodan Chong, Cuibo Liu, Yi Huang, Chenqi Huang, Bin Zhang

**Affiliations:** 1 Department of Chemistry, Institute of Molecular Plus, School of Science, Tianjin University, Tianjin 300072, China; 2 Tianjin Key Laboratory of Molecular Optoelectronic Sciences, Collaborative Innovation Center of Chemical Science and Engineering, Tianjin 300072, China

**Keywords:** azoxy, azo- and amino-aromatics, electrosynthesis, metal phosphides, nitroarenes, selectivity

## Abstract

Azoxy-, azo- and amino-aromatics are among the most widely used building blocks in materials science pharmaceuticals and synthetic chemistry, but their controllable and green synthesis has not yet been well established. Herein, a facile potential-tuned electrosynthesis of azoxy-, azo- and amino-aromatics via aqueous selective reduction of nitroarene feedstocks over a CoP nanosheet cathode is developed. A series of azoxy-, azo- and amino-compounds with excellent selectivity, good functional group tolerance and high yields are produced by applying different bias input. The synthetically significant and challenging asymmetric azoxy-aromatics can be controllably synthesized in moderate to good yields. The use of water as the hydrogen source makes this strategy remarkably fascinating and promising. In addition, deuterated aromatic amines with a high deuterium content can be readily obtained by using D_2_O. By pairing with anodic oxidation of aliphatic amines to nitriles, synthetically useful building blocks can be simultaneously produced in a CoP||Ni_2_P two-electrode electrolyzer. Only 1.25 V is required to achieve a current density of 20 mA cm^−2^, which is much lower than that of overall water splitting (1.70 V). The paired oxidation and reduction reactions can also be driven using a 1.5 V battery to synthesize nitrile and azoxybenzene with good yields and selectivity, further emphasizing the flexibility and controllability of our method. This work paves the way for a promising approach to the green synthesis of valuable chemicals through potential-controlled electrosynthesis.

## INTRODUCTION

Azoxy-, azo- and amino-compounds are important organic molecules that have attracted much interest within the chemistry community due to their unique properties arising from the N−N−O and N=N moieties in azoxy- and azo-molecules and the vital applications of amines for the industrial production of dyestuffs, pharmaceuticals, and other chemicals [1,2]. A substantial effort has been devoted to their synthesis in a profitable and efficient manner, where selective reduction of nitro feedstocks outperforms other strategies due to their wide commercial availability and ease of implementation [3–5]. However, the nitro reduction proceeds via multiple electron-proton coupled steps [6–8]. This poses practical difficulties in controlling the degree of reduction against the production of thermodynamically favorable, over-hydrogenated amines [9–11]. Thus, the synthesis of highly valuable azoxy and azo compounds remains a critical challenge. Several strategies have been proposed for selective synthesis of azoxy and azo compounds by changing the adsorption/desorption energy of some reaction intermediates (such as nitrosobenzene and phenylhydroxylamine) and/or the reactive hydrogen (*H) to the catalysts. However, most of these strategies have been focused on the modifications of catalysts [12–15] or use of light with different wavelengths [16] to realize different product selectivity, inevitably suffering from tedious modifications or requirements for the use of specific light sources. High pressure H_2_ and other expensive and hard-to-control donors (such as NH_2_NH_2_·H_2_O, NaBH_4_) remain the primary hydrogen sources, posing serious safety risk and environmental concerns and leading to the poor functional group compatibility of the current methods. Thus, it is highly desirable to develop a facile, efficient and sustainable method to achieve the synthesis of azoxy-, azo- and amino-compounds through the reduction of nitro feedstocks in a controllable manner.

Recently, electrochemical transformation has offered a tunable and efficient alternative in synthetic chemistry due to its precise control by the external potential or current [17–25]. Electrons can act as green reductants to drive chemical reactions. By designing appropriate catalytic materials, the chemoselectivity can be well tuned by adjusting the potential or current input, altering the concentration and types of reactive intermediates at the electrode surface and finally determining the product distributions. For example, in the electrochemical CO_2_ reduction reactions (CO_2_RR) on Cu electrodes, generally, 2e^−^ products (H_2_, CO and HCOOH) are preferred at lower overpotentials, whereas higher e^−^ products (CH_4_: 8e^−^ and C_2_H_4_: 6e^−^) are formed at higher overpotentials [26–28]. Inspired by the fundamental understanding of CO_2_RR and the electrochemical hydrogen evolution reaction (HER), it is intriguing to develop an electrochemical pathway to reduce nitro substrates specifically to azoxy-, azo- and amino-products in aqueous solution at different electrochemical conditions by balancing the nitro reduction with the competitive HER. In this case, the use of water as the sole hydrogen source will provide not only a safer, cleaner and more sustainable way to traditional nitro reductions using H_2_, metal hydrides, hydrazine hydrate, silane, and other reagents [3–15] but also the synthesis of value-added deuterated amines (–ND_2_) with potential utilization for deuterated drugs with enhanced therapeutic effect and LC/MS analysis with increased sensitivity, ionization efficiency, and chromatographic performance [29–31]. The latter can be readily realized by using D_2_O to replace H_2_O, in sharp contrast to previous –ND_2_ synthesis using expensive and hard-to-obtain deuterated D_2_, LiAlD_4_ and DCl [32,33]. Currently, the electrochemical reduction of nitro compounds relies on the use of noble metal electrodes and suffers from low conversion, narrow substrate scopes and poor selectivity [34,35]. Furthermore, the operational conditions in acidic solutions (such as HClO_4_) make it impractical and challenging to synthesize –ND_2_ with a high deuteration content [36,37].

**Figure 1.**
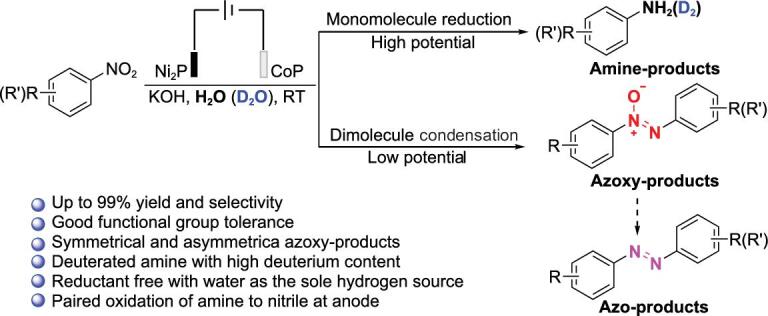
Highly selective electrochemical reduction of nitroarenes to azoxy-, azo- and amino-aromatics over a CoP cathode.

Herein, we reported a highly efficient aqueous electrosynthesis of azoxy-, azo- and amino-aromatics by selective reduction of nitro substrates over a cobalt phosphide (CoP) nanosheet cathode (Fig. 1). The product selectivity can be flexibly controlled by adjusting the applied potentials to modulate the competition between HER and nitro reduction. The use of CoP as cathode and water as solvent avoids the excessive reduction of nitro aromatics to cyclohexylamine [38]. Deuterated amino compounds (–ND_2_) can be efficiently fabricated by using D_2_O as the hydrogen source.

## RESULTS AND DISCUSSION

### CoP nanosheet electrode

**Figure 2.**
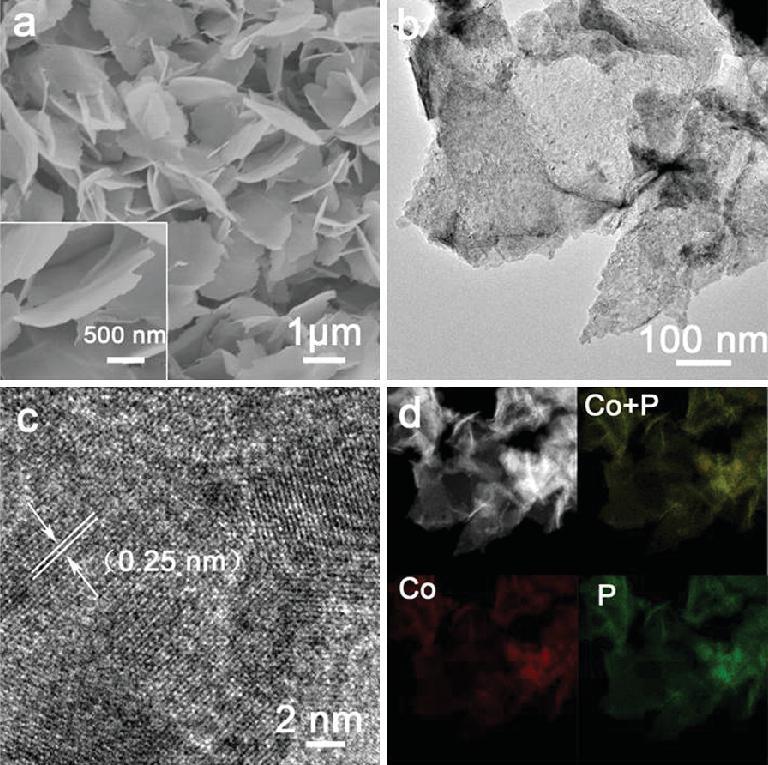
(a) SEM (inset: enlargement), (b) TEM, (c) HRTEM, and (d) EDS elemental mapping images of CoP nanosheets.

Co-related catalysts are widely used in catalysis due to their low cost, abundance and high selectivity [39–41]. The moderate Co-*H formation energy makes it highly suitable for use in hydrogenation and HER through the H_2_ dissociation and H-H combination. CoP nanosheets have shown electrocatalytic performance comparable to that of Pt-based materials due to their abundance of exposed catalytically active sites, high catalytic activity and excellent stability toward electrochemical HER [42–45]. Thus, CoP nanosheets were chosen as the model electrocatalyst to evaluate our conceptual strategy on potential-tuned selective electrosynthesis of azoxy-, azo- and amino-aromatics. The CoP nanosheet electrode was prepared by direct phosphating of cobalt hydroxide precursors [42]. Scanning electron microscopy (SEM) images show that the nanosheet arrays are anchored on nickel foam (Fig. 2a). The transmission electron microscopy (TEM) image confirms the nanosheet morphology (Fig. 2b). The high-resolution TEM (HRTEM) image shows a lattice spacing of 0.25 nm indexed to the (111) plane of orthorhombic CoP (Fig. 2c). All diffraction peaks in the X-ray diffraction (XRD) pattern can be indexed to orthorhombic CoP (JCPDS No. 29–0497) (Supplementary Fig. 1a). X-ray photoelectron spectroscopy (XPS) spectra also show the characteristic peaks of CoP (Supplementary Fig. 1b-d), as observed in [42]. Additionally, the energy-dispersive X-ray spectroscopy (EDS) elemental mapping images indicate that the Co and P elements are uniformly distributed (Fig. 2d). These results suggest the successful synthesis of the CoP nanosheet electrode.

## Selective electrochemical reduction of nitroarenes to azoxy-, azo- and amino-aromatics

**Figure 3.**
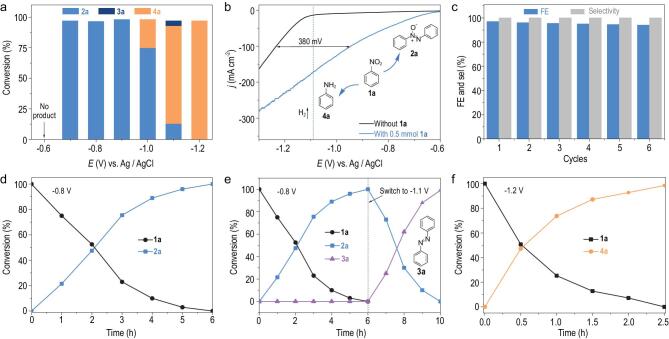
(a) Cathodic products from the electrochemical reduction of **1a** over CoP obtained at different potentials. (b) LSV curves of CoP at a scan rate of 5 mV s^−1^ in 1.0 M KOH with and without 0.5 mmol of **1a**. (c) Cycle-dependent selectivity and FEs of **2a** over CoP at −0.8 V vs. Ag/AgCl. (d-f) Time-dependent conversion plots for the electrochemical reduction of **1a** into **2a, 3a** and **4a** over a CoP cathode, respectively, in 1.0 M KOH at different potentials.

The electrochemical reduction of nitro substrate was first applied to the selective synthesis of azoxy-aromatic compounds, which is the most challenging synthetic task among azoxy-, azo- and amino-aromatics using reported methods [4,10–12,16]. The reaction was implemented in a 1.0 M KOH aqueous solution using a standard three-compartment electrochemical cell. Not that, all the electrochemical measurements were carried out under ambient atmosphere. Nitrobenzene (**1a**) was chosen as the model substrate. Different potentials were first applied ranging from −0.6 V to −1.2 V (vs. Ag/AgCl) to investigate the product distributions through long-term chronoamperometry with 1.0 mmol of **1a** (Fig. 3a and Supplementary Table 1). As expected, similar to CO_2_RR, azoxybenzene (**2a**) with 99% selectivity and full conversion of **1a** was realized at less negative potentials, whereas aniline (**4a**) (6e^−^) dominated at high reductive voltages. Although we were unable to directly synthesize azobenzene (**3a**) by tuning the potential, post-transformation of **2a** at higher reductive voltages led to the formation of **3a** with high yield and selectivity, as will be discussed below. Figure 3b shows the linear sweep voltammetry (LSV) curves of nitro reduction and HER over a CoP cathode with and without 1.0 mmol of **1a**, respectively. For HER, the onset potential is approximately −1.10 V (vs. Ag/AgCl), and H_2_ bubbles can be clearly observed when the potential reaches −1.12 V. Upon the addition of **1a**, the current density starts to increase from −0.6 V and the H_2_ bubbles clearly appear until the potential reaches −1.12 V. These results show that the reduction of **1a** occurs preferentially at more positive potentials than HER due to its more electron-deficient property. Time-depended transformations reveal that the reaction can be finished within 6 h at −0.8 V (Fig. 3d). Using Ni_2_P or FeP as the cathodes, inferior yield and selectivity were observed, which may be ascribed to the moderate adsorption/desorption of highly active *H on the CoP surface [46]. Pure Ni foam (NF) was almost useless. All of the control experiments indicated the remarkable superiority of CoP in the selective conversion of **1a** into **2a** (Supplementary Fig. 2). In addition, no detectable chemical transformation occurred without electricity, suggesting the reduction reaction is mediated by electro-catalysis. The use of CH_3_CN/H_2_O (25 mL/40 μL) and CH_3_OH as the solvents under otherwise identical conditions led to poor yields and selectivity (Supplementary Fig. 3a), revealing the important role of water in controlling and promoting the selective nitro reduction. Finally, an undivided cell was adopted for more convenient production. However, **2a** is delivered in lower yield due to the re-oxidation of the intermediates or azoxy products at the anode (Supplementary Fig. 3b).

To validate the universality of this electrochemical synthesis of azoxy aromatic compounds, a variety of nitroarenes were examined (Supplementary Fig. 4). The results in Table 1 demonstrate that this strategy has excellent functional-group tolerance. The nitro substrates with the electron-donating or -withdrawing groups on the aryl ring all work well to give the corresponding azoxy-aromatic products with high conversion yields and excellent selectivity. No obvious decreases in reaction efficiency are observed when installing the -F and -Cl groups at the *ortho*-position of the nitro group (Table 1, **2j** and **2k**), indicating an inessential steric hindrance. Due to the mild reaction conditions, the quite fragile C-Br and C=C bonds are retained well, which is challenging in the traditional hydrogenation methods (Table 1, **2l-m**), providing good opportunities for the subsequent synthesis of more complex molecules. Furthermore, this method can be applied to gram-scale synthesis with no obvious decrease in the reaction yields (Supplementary Fig. 5). All of these results prove that our established strategy is versatile and efficient for the synthesis of azoxy-aromatic compounds.

**Table 1.** Electrochemical reduction of nitroarenes to symmetrical and asymmetrical azoxy-aromatics over a CoP cathode.

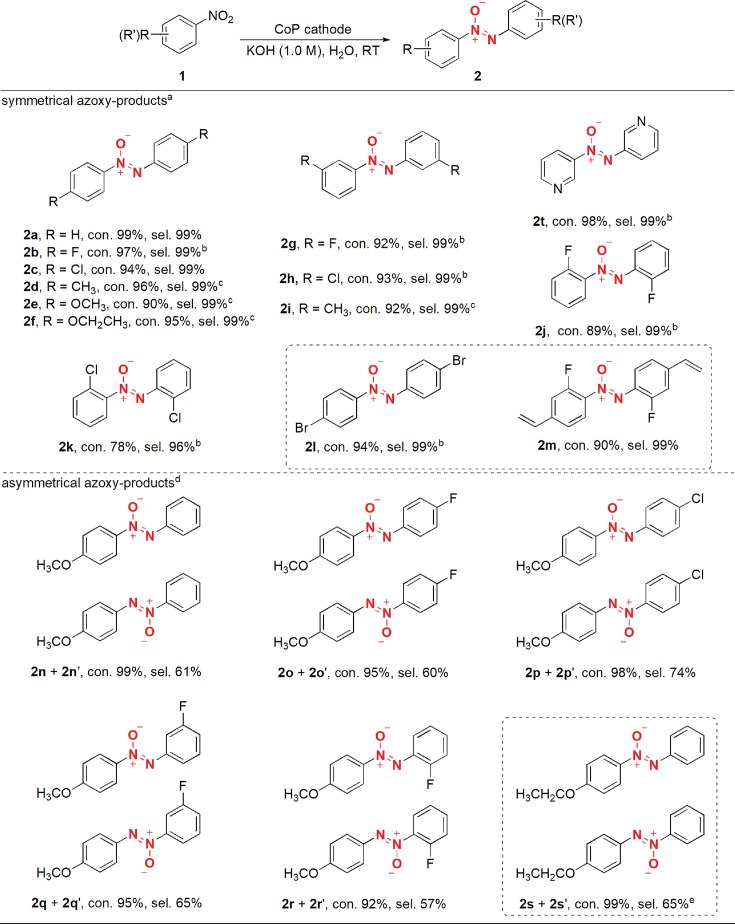

^a^Nitro substrates (0.5 mmol), CoP (working area: 1 cm^2^), 1.0 M KOH (25 mL), room temperature, −0.75 V vs. Ag/AgCl. Con. is the abbreviation for conversion and sel. for selectivity. The con. and sel. are determined by gas chromatography. ^b^−0.7 V vs. Ag/AgCl was used. ^c^−0.8 V vs. Ag/AgCl was used. ^d^*p*-methoxynitrobenzene (0.5 mmol), other nitro substrates (1.5 mmol). ^e^*p*-ethoxynitrobenzene (0.5 mmol), **1a** (1.5 mmol), −0.8 V vs. Ag/AgCl.

Importantly, asymmetrically substituted azoxy-aromatic compounds that are difficult to be synthesized by the present methods can be constructed using this controllable electrochemical method under the optimized reaction conditions. Note that, *p*-methoxynitrobenzene is used as 1.0 equivalent, while the other nitro substrates are in excess for enhanced yields of the cross-coupling products due to the faster kinetics of the electron-withdrawing aryl substituents (Table 1, **2o** + **o’** to **2s** + **s’**). Nearly complete conversion of *p*-methoxynitrobenzene with moderate yields of the asymmetric products was obtained. The slightly low yield for each case is ascribed to the competitive role of the homocoupling reaction, but its yield is still much higher than the reported yield values [47]. Notably, our method can provide a convenient approach to the efficient synthesis of *p*-azoxyphenetole (**2s** + **s’**), which is an important nematic liquid crystal [48].

Azobenzenes are regarded as one of the largest and most commonly used organic dyes, and their selective synthesis has been a long-standing challenge [2,5,13–16,49]. By using our electrochemical method, azobenzene can be efficiently synthesized through a one-pot two-step procedure involving the first formation of azoxy products at a less negative bias (−0.8 V vs. Ag/AgCl) and their subsequent deep reduction to azo at the more negative potentials (−1.1 V vs. Ag/AgCl) (Fig. 3e and Supplementary Fig. 6). This one-pot two-step production of azo-aromatics from nitro reduction further demonstrates the good controllability of this potential-tuned strategy. We note that the amino product could not be detected during this procedure. This proves that the formation of amine via mono-molecule reduction is not involved in the formation of an azo-intermediate (Supplementary Table 1 and Supplementary Fig. 7). Some representative examples with high yields and selectivity are displayed in Table 2, providing a new alternative to the current synthesis methods of azo-compounds.

**Table 2.** Electrochemical reduction of nitroarenes to azo-aromatics over a CoP cathode^a^.

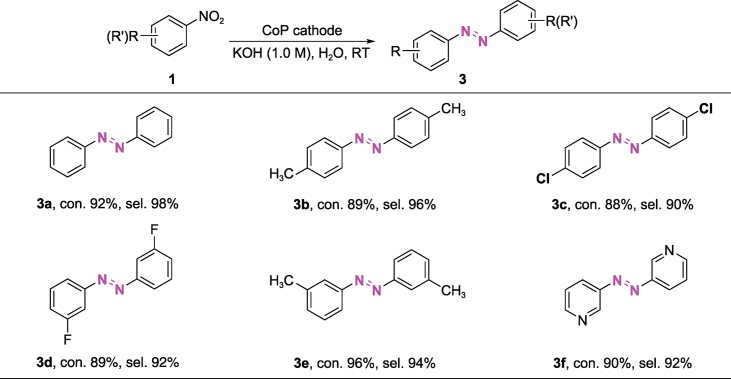

^a^Nitro substrates (0.5 mmol), CoP (working area: 1 cm^2^), 1.0 M KOH (25 mL), room temperature, −0.75 V and then turned to −1.1 V vs. Ag/AgCl. Con. is the abbreviation for conversion and sel. for selectivity. The con. and sel. are determined by gas chromatography.

Amino aromatic compounds are a class of essential and versatile building blocks for drug synthesis, material preparations and catalyst designs/modifications [1,3,39,40]. Our method was also quite applicable to the rapid synthesis of amino aromatic compounds within 2.5 h (Fig. 3f). As observed from the data presented in Table 3, a variety of nitro substrates with electro-donating and electron-withdrawing groups on the *ortho*, *meta* or *para* positions on the aryl ring were selectively transformed into the corresponding aromatic amines with yields in the 94–98% range (**4a**-**k** and **4p**-**s**). The multi-substituted and sterically constrained substrates worked smoothly to provide **4l** and **4m** in good yields. No decreases in the yields were observed for *o*-dinitrobenzene and 3-nitropyridine (**4n**-**o**). We note that the highly fragile -C–Br, -C=O and -C=C groups were well retained to the products without their simultaneous hydrogenation and without the detection of coupling or condensation products between -C–Br or -C=O and -NH_2_; these features are always challenging to obtain by other methods [50]. These unexpected chemoselective properties may be ascribed to both the electron-deficient effect and the preferred adsorption of the -NO_2_ group on the surface of CoP compared to the other groups [51,52]. Benefiting from this electrocatalytic method, deuterated aromatic amines with high deuterium content are easily synthesized (**4w**), demonstrating that this electroreductive strategy has remarkable advantages relative to the traditional approaches in terms of the operation, cost and sustainability.

**Table 3.** Electrochemical reduction of nitroarenes to amino-aromatics over a CoP cathode^a^.

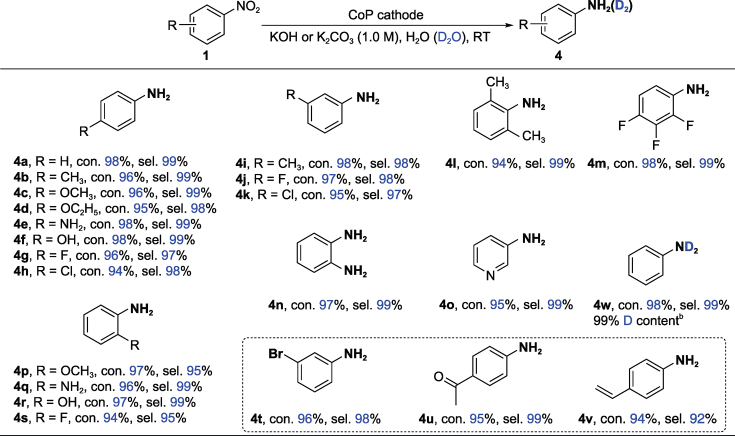

^a^Nitro substrates (0.5 mmol), CoP (working area: 1 cm^2^), 1.0 M KOH (25 mL), room temperature, −0.9 V ∼ −1.2 V vs. Ag/AgCl. Con. is the abbreviation for conversion and sel. for selectivity. The con. and sel. are determined by gas chromatography. ^b^1.0 M K_2_CO_3_ was used as the electrolyte.

Stability and reusability are essential properties for evaluating the potential utility of a catalyst. At a constant potential of −0.8 V vs. Ag/AgCl, one piece of catalyst was reused to reduce 2.0 mmol of nitrobenzene for six times. High Faradaic efficiencies (FEs) and 99% yield of **2a** can be maintained for at least six cycle use, revealing good durability of the CoP cathode for such electrochemical reduction process (Fig. 3c). No obvious changes in the used CoP cathode were observed by SEM and XPS, suggesting the good stability of the cathode (Supplementary Figs. 8 and 10). The diffraction peaks located at 31.774, 36.413, 46.282, 48.363, 56.489 and 76.081 of used CoP could be indexed to the (011), (111), (112), (211), (301) and (222) plane of orthorhombic CoP (JCPDS No. 29–0497), which is consistent well with the fresh one (Supplementary Fig. 9).

**Figure 4.**
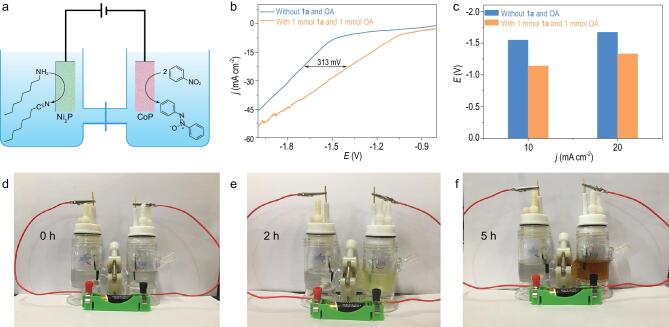
(a) Illustration for coupling cathodic electro-reductive synthesis of **2a** with anodic conversion of octylamine into octylnitrile in a CoP||Ni_2_P electrolyzer. (b) LSV curves and (c) comparison of potential for achieving benchmark current densities in a CoP||Ni_2_P electrolyzer at a scan rate of 5 mV s^−1^ in 1.0 M KOH before and after adding 1.0 mmol of **1a** to cathode chamber and 1.0 mmol of octylamine to anode chamber. (d-f) Time-dependent photos of a CoP||Ni_2_P electrolyzer setup driven by 1.5 V battery for both anodic oxidation of octylamine and cathodic reduction of **1a** to **2a**.

Finally, to enhance the practicability of our method, a two-electrode configuration using CoP as the cathode and Ni_2_P as the anode was assembled in a separated CoP||Ni_2_P cell. To reduce the total energy input, oxidation of the thermodynamically more favorable organic compounds (e.g. alcohols, amines) was used to replace the kinetically sluggish oxygen evolution reaction at the anode [53–58]. Here, cathodic electrochemical reduction of **1a** and anodic conversion of octylamine were selected as the model cases (Fig. 4a). As depicted in Fig. 4b and c, the required cell voltage for achieving the current density of 20 mA cm^−2^ is only 1.25 V (vs. counter electrode) after adding 1.0 mmol of **1a** and 1.0 mmol of octylamine in the cathode and anode chambers, respectively. This cell voltage is much lower than that required for overall water splitting (1.70 V). Furthermore, a long-term electrolysis at −1.2 V over a CoP||Ni_2_P electrolyzer was performed to quantify the products of the cathode and anode chambers. Both **2a** and octylnitrile with nearly 100% selectivity were obtained (Supplementary Fig. 11). Impressively, this two-electrode configuration can be used at the gram scale (∼2 g) under standard conditions with comparable conversion and selectivity, indicating the potential utility of this two-electrode electrolyzer for both the nitro reduction and amine oxidation that cannot be achieved by traditional methods. Finally, as revealed in Fig. 3a and Supplementary Fig. 12, **2a** can be obtained with ∼99% yield and FEs at the potentials ranging from −0.65 to −0.95 V, leading to the possibility of distributed synthesis of azoxy using either a photo-voltaic-integrated solar panel or a 1.5 V battery (Fig. 4d and e). Comparable yields and selectivities for both **2a** and nitrile products were ultimately obtained using the two-electrode electrolyzer, demonstrating the remarkable feasibility and practicability of our method.

As mentioned above, the nitro reduction proceeds via multiple electron-proton coupled steps. Catalyst engineering to alter the adsorption/desorption energy of some reaction intermediates to the catalysts is a favorable method for tuning the product selectivity [12–15]. For our electrochemical method, potential tuning can be used to control the intermediates. It is well-accepted that the formation of azoxy- and azo-products involves the condensation of two intermediates, namely, nitrosobenzene and phenylhydroxylamine [8]. Additionally, 1.0 M KOH is essential because it will suppress the unwanted hydrogenation of the two intermediates due to the trace amount of protons at low potentials. At a higher reductive potential, more active *H will be generated at the surface of CoP via water splitting, trapping phenylhydroxylamine to form aniline and inhibiting the condensation of nitrosobenzene and phenylhydroxylamine (Supplementary Fig. 7). Cobalt in CoP is known to have a partial positive charge (δ^+^) [59], favoring adsorption of nitrobenzene and facilitating the reduction reactions.

## CONCLUSIONS

In summary, we demonstrated a potential-tuned strategy for aqueous selective reduction of nitro substrates to azoxy-, azo- and amino-aromatics over a CoP nanosheet cathode. A variety of products bearing different functional groups were efficiently synthesized. In particular, the highly fragile -C-Br, -C=O and -C=C bonds were retained well for the synthesis of aromatic amines under our conditions. This good chemoselectivity is attributed to the electron-deficient effect and the preferred adsorption of the -NO_2_ group on CoP. Additionally, our protocol can also achieve the synthesis of unique asymmetric azoxy-aromatic compounds that are challenging for the traditional methods. Compared to the methods that rely on catalyst modifications to realize product selectivity, our potential-tuned strategy is more convenient to operate to tune the products selectivity at a gram scale for practical applications in industrial production. The adoption of water as the sole hydrogen source also provides a convenient method for producing deuterated amino aromatics with high deuterated efficiency. Furthermore, pairing with anodic oxidation of aliphatic amines, gram-scale production of azoxybenzene and octylnitrile can be simultaneously achieved with high efficiency in a two-electrode electrolyzer. Impressively, the paired reaction can be operated using a 1.5 V battery with good yield and selectivity, paving the way for low-cost and efficient production of both azoxy-aromatic and aliphatic nitrile. This potential-tuned strategy by using water as the hydrogenation source with good product selectivity can find wide applications in other types of electrochemical reduction reactions for controllable and green synthesis.

## METHODS

### Synthesis of CoP nanosheet electrode

Firstly, a piece of nickel foam (NF) (3 cm × 1 cm × 0.1 cm) was ultrasonicated with acetone, water and 3.0 M HCl aqueous solution for 5 min, respectively. Then, the NF was rinsed with distilled water (DIW) and anhydrous alcohol. Lastly, the NF was quickly dried under ambient conditions. The CoP electrode was prepared through modifying the reported method [42]. Typically, a *α*-Co(OH)_2_ precursor was electrodeposited onto the fresh-treated NF (*α*-Co(OH)_2_/NF) at −1.0 V vs. SCE (saturated calomel electrode) in 0.025 M Co(NO_3_)_2_ solution. Then, a piece of *α*-Co(OH)_2_/NF and NaH_2_PO_2_ (0.1 g) were put at two separate position in a porcelain boat with NaH_2_PO_2_ at the upstream side of the furnace. Subsequently, the sample was heated at 300°C for 2 h in static Ar atmosphere, and then naturally cooled to ambient temperature under Ar atmosphere.

## Selective electrochemical reduction of nitroarenes to azoxy-aromatics

In 25 mL aqueous electrolyte (1.0 M KOH) containing 0.5 mmol of nitroarenes, the linear sweep voltammetry (LSV) curves tests with the scan rate of 5 mV s^−1^ ranging from −0.6 to −1.3 V vs. Ag/AgCl were implemented to study the onset reductive potentials of nitroarenes. To achieve azoxy-aromatics products, chronoamperometry was carried out at a given constant potential ranging from −0.7 to −0.8 V vs. Ag/AgCl in 1.0 M KOH solution.

## Selective electrochemical reduction of nitroarenes to azo-aromatics

To achieve azo aromatics products, chronoamperometry was firstly carried out at a low potential ranging from −0.7 to −0.8 V vs. Ag/AgCl in 25 mL aqueous electrolyte (1.0 M KOH) containing 0.5 mmol of nitroarenes to produce azoxy-aromatics first. After nitroarenes were completely transferred to azoxy-aromatics, the constant potential is quickly swift to a high potential (e.g. −1.1 V vs. Ag/AgCl for azobenzene) to synthesize azo-aromatics.

## Selective electrochemical reduction of nitroarenes to amino-aromatics

To achieve amino aromatics products, firstly long-term chronoamperometry was carried out at more negative constant potentials ranging from −0.9 to −1.2 V vs. Ag/AgCl in 25 mL aqueous electrolyte (1.0 M KOH) containing 0.5 mmol of nitroarenes.

## Characterization

The scanning electron microscopy (SEM) images were taken with a Hitachi S-4800 scanning electron microscope (3 kV). The transmission electron microscopy (TEM) images, the energy-dispersive X-ray spectroscopy (EDS) elemental mapping images and higher-resolution transmission electron microscopy (HRTEM) images were obtained with JEOL-2100F system equipped with EDAX Genesis XM2. The X-ray diffraction (XRD) patterns of the products were recorded with Bruker D8 Focus Diffraction System using a Cu K*α* source (λ = 0.15406 nm). X-ray photoelectron spectroscopy (XPS) measurements were performed on a photoelectron spectrometer using Al K*α* radiation as the excitation source (PHI 5000 VersaProbe). All the peaks were calibrated with C 1 s spectrum at binding energy of 284.8 eV. The NMR spectra were recorded on Varian Mercury Plus 400 instruments at 400 MHz (^1^H NMR) and 101 MHz (^13^C NMR). Chemical shifts were reported in parts per million (ppm) down field from internal tetramethysilane. Multiplicity was indicated as follows: s (singlet), d (doublet), t (triplet), q (quartet), mm (multiplet), dd (doublet of doublet), br (broad). Coupling constants were reported in hertz (Hz). The gas chromatograph-mass spectrometer (GC-MS) was carried out with TRACE DSQ. The gas chromatograph (GC) was measured on Aglient 7890A with thermal conductivity (TCD) and flame ionization detector (FID). The injection temperature was set at 300°C. Nitrogen was used as the carrier gas at 1.5 mL min^−1^. All reported data are averages of experiments performed at least thrice.

## Electrochemical measurements

Electrochemical measurements were carried out in a standard three-compartment electrochemical cell consisting of a working electrode, a Pt plate counter electrode, and a Ag/AgCl (1.0 M KCl) reference electrode, and all the potentials in this work are referred to Ag/AgCl unless otherwise stated. Linear sweep voltammetry (LSV), chronoamperometry was performed using an electrochemical workstation (CHI 660D, Chenhua, Shanghai). The solution of 1.0 M KOH (pH = 13.6) was employed as electrolyte. The NF with catalyst samples directly grown on the surface was used as the working electrode with exposed surface area of 1.0 cm^2^. The electrochemical hydrogen evolution reaction and electrocatalytic reduction reactions of nitroarenes experiments were conducted in 25 mL of 1.0 M KOH solution with and without 1.0 mmol of nitroarenes. The electrochemical OER experiment was also conducted in 25 mL of 1.0 M KOH solution. For two-electrode electrolysis, CoP and Ni_2_P were employed as cathode and anode, respectively. The scan rates of LSV curves were 5 mV s^−1^.

The stability tests of CoP electrode for **1a** reduction were evaluated by chronoamperometry at −0.8 V in 25 mL of 1.0 M KOH solution using 2.0 mmol of **1a**. All experiments were carried out at room temperature (RT, 25 ± 1°C) under ambient atmosphere.

## Quantitative analysis of reduction and oxidative products

To analyze the products of nitrobenzene reduction and calculate the corresponding FEs, 25 mL of electrolyte solution was extracted with acetic ether after chronoamperometry testing with ∼289 C of total passing charge. Simultaneously, in the two-electrode system, the generated octylnitrile at anode was also collected. The extracted products were confirmed by the comparisons of their GC retention time and mass spectra. Yields were determined by GC analysis. Here, we take azoxy-compounds and octylnitrile as the examples to show the calculated equations of the cathodic and anodic products, respectively. For azo- and amino-products, the calculated equations are similar to those of azoxy-compounds.

The conversion (%) and selectivity (%) of the formed octylnitrile were calculated using equations (1) and (2), respectively:


(1) \begin{eqnarray*} &&\mathrm{Conversion}\ \left(\%\right)\nonumber\\ &&\quad=\frac{\mathrm{mol}\ \mathrm{of}\ \mathrm{the}\ \mathrm{consumed}\ \mathrm{octylamine}}{\mathrm{mol}\ \mathrm{of}\ \mathrm{the}\ \mathrm{added}\ \mathrm{octylamine}}\times 100\%,\nonumber\\ \end{eqnarray*}

(2) \begin{eqnarray*} &&\mathrm{Selectivity}\left(\%\right)\nonumber\\ &&\quad=\frac{\mathrm{mol}\ \mathrm{of}\ \mathrm{the}\ \mathrm{formed}\ \mathrm{octylnitrile}}{\mathrm{mol}\ \mathrm{of}\ \mathrm{the}\ \mathrm{consumed}\ \mathrm{octylamine}} &&\times 100\%.\nonumber\\ \end{eqnarray*}

The FEs for the formation of azoxy-aromatic compounds on CoP cathode and octylnitrile on Ni_2_P anode were calculated using the equation (3) and (4), respectively:


(3) \begin{eqnarray*} \mathrm{FE}\left(\%\right)\nonumber\\ = \frac{\mathrm{mol}\ \mathrm{of}\ \mathrm{the}\ \mathrm{formed}\ \mathrm{azoxy}\ \mathrm{compounds}}{\mathrm{The}\ \mathrm{total}\ \mathrm{passed}\ \mathrm{charge}/6\mathrm{F}}\,{\times}\,100\%,\nonumber\\ \end{eqnarray*}

(4) \begin{eqnarray*} &&\mathrm{FE}\left(\%\right)\nonumber\\ &&\quad =\frac{\mathrm{mol}\ \mathrm{of}\ \mathrm{the}\ \mathrm{formed}\ \mathrm{octylnitrile}}{\ \mathrm{The}\ \mathrm{total}\ \mathrm{passed}\ \mathrm{charge}/4\mathrm{F}}\times 100\%, \nonumber\\ \end{eqnarray*}

where *F* is the Faraday constant (96 485 C mol^−1^).

A theoretical charge for the complete reduction of nitroarenes (1.0 mmol) to azoxy aromatic compounds (0.5 mmol): (1.0 × 10^−3^ mol) ×3 × (6.02 × 10^23^ mol^−1^) × (1.6 × 10^−19^ C) =∼289 C.

A theoretical charge for the complete oxidation of octylamine (1.0 mmol) to octylnitrile (1.0 mmol): (1.0 × 10^−3^ mol) × 4 × (6.02 × 10^23^ mol^−1^) ×(1.6 × 10^−19^ C) = ∼385 C.

## Supplementary Material

nwz146_Supplemental_File
