# Supplementary material for: Potential-tuned selective electrosynthesis of azoxy-, azo- and amino-aromatics over a CoP nanosheet cathode
Source: Natl Sci Rev. 2019 Oct 1;7(2):285–95. doi: 10.1093/nsr/nwz146 (PMC8288891; doi:10.1093/nsr/nwz146)
Supplement: nwz146_Supplemental_File [file nwz146_supplemental_file.docx]

Supplementary Information for

**Potential-Tuned Selective Electrosynthesis of Azoxy-, Azo- and Amino Aromatics over a CoP Nanosheet Cathode**

Xiaodan Chong^1#^, Cuibo Liu^1#^, Yi Huang^1^, Chenqi Huang^1^ & Bin Zhang^1,2^*

^1^Department of Chemistry, Institute of Molecular Plus, School of Science, Tianjin University, Tianjin 300072, China

^2^Tianjin Key Laboratory of Molecular Optoelectronic Sciences, Collaborative Innovation Center of Chemical Science and Engineering, Tianjin 300072, China

^#^These authors contributed equally to this work

*e-mail: bzhang@tju.edu.cn

**Supplementary Figure 1.** a) XRD pattern, b) XPS survey spectrum and c-d) high-resolution XPS spectra of the fresh-prepared CoP: c) Co 2p, d) P 2p. The peaks at 793.5 eV, 798.2 eV and one satellite peak at 803.1 eV are attributed to Co 2p_1/2_. The peaks at 778.7 eV, 781.8 eV and one satellite peak at 786.2 eV are assigned to Co 2p_3/2_. Similarly, the two peaks at 129.4 eV and 130.2 eV are assigned to P 2p_3/2_ and P 2p_1/2_ of CoP, respectively. The peak at 133.8 eV is attributed to the P-O may come from the phosphate-like P.^[1-4]^

**Supplementary** **Figure 2.** Comparison of electrochemical reduction of **1a** over Ni foam, CoP, FeP and Ni_2_P cathodes at the potential of -0.80 V vs. Ag/AgCl in 1.0 M KOH aqueous solution.

**Supplementary Figure 3.** a) Electrochemical reduction of **1a** over a CoP cathode at the potential of -0.8 V vs. Ag/AgCl in different solvents, b) Electrochemical reduction of **1a** to **2a** over a CoP cathode at the potential of -0.8V vs. Ag/AgCl in divided and undivided cell in 1.0 M KOH aqueous solution.

**Supplementary Figure 4.** LSVs curve of a CoP nanosheet cathode in 1.0 M KOH without and with various nitroarenes (0.5 mmol): a) *p*-nitrofluorobenzene, b) *p*-chloronitrobenzene, c) *p*-nitrotoluene, d) *p*-nitroanisole, e) *p*-nitrophenetole, f) *p*-nitroacetophenone, g) nitrostyrene, h) *p*-nitroaniline, i) *p*-nitrophenol, j) *p*-bromonitrobenzene, k) *m*-fluoronitrobenzene, l) *m*-chloronitrobenzene, m) 3-nitrotoluene, n) *m*-bromonitrobenzene, o) *o*-fluoronitrobenzene, p)  2,3,4-trifluoronitrobenzene, q) *o*-nitroanisole, r) *m*-nitropyridine, s) *o*-nitroaniline, t) *o*-nitrophenol, u) 1,3-dinitrobenzene, v) 2,6-dimethylnitrobenzene, w) nitrobenzene and *o*-fluoronitrobenzene, x) nitrobenzene and *p*-nitrophenetole, y) nitrobenzene and *p*-nitroanisole, z) *p*-fluoronitrobenzene and *p*-nitroanisole, a_1_) *p*-chloronitrobenzene and *p*-nitroanisole, a_2_) *m*-fluoronitrobenzene and *p*-nitroanisole. Scan rate: 5 mV s^-1^.

**Supplementary Figure 5.** Gram-scale preparation of different substituted azoxy-aromatic products.

Scale-up synthesis to azxoy-products bearing different functional groups with no obvious decrease of conversion yield and selectivity reveals the practical utilization for potential industrial productions.

**Supplementary Figure 6.** Conversion and selectivity of highly electrochemical reduction of **1a** to **2a** at -0.8 V vs. Ag/AgCl, and **2a** to **3a** when voltage turned to -1.1 V vs. Ag/AgCl over a CoP cathode in 1.0 M KOH solution.

**Supplementary Table 1.** Control experiments for electrochemical reduction of different reactants.

All the reduction reactions were carried out in a standard three-compartment electrochemical cell under ambient atmosphere. Nitrobenzene can be only reduced to azoxybenzene at the less negative potential (-0.8 V vs. Ag/AgCl) (entry 1), while at the more negative potential (-1.2 V vs. Ag/AgCl), aniline is delivered as the solo product (entry 2). Although azobenzene cannot be achieved via adjusting of the reductive potential directly, it can be selectively produced at -1.2 V by using azoxybenzene as the starting material (entry 3). Not that, aniline cannot be detected either azoxybenzene or azobenzene was used as the starting material at the -1.2 V vs. Ag/AgCl in 1.0 M KOH solution. All the above results revealed that different reaction pathways ware involved for the formation of azoxy-, azo- and amino-aromatics under different reduction potentials. A possible mechanism was proposed in **Supplementary Figure 7**.

**Supplementary Figure 7.** Proposed mechanism of highly selective electrochemical reduction of nitrobenzene over a CoP cathode in 1.0 M KOH solution.


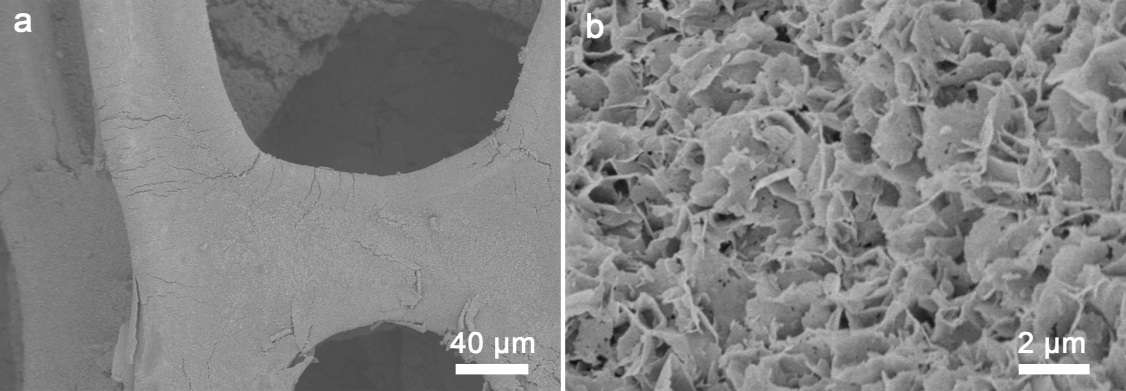


**Supplementary Figure 8.** a, b) SEM images of CoP nanosheet cathode after six cycles for highly selective electrochemical reduction of **1a** to **2a**.

**Supplementary Figure 9.** XRD patterns of CoP nanosheet cathode after six cycles for highly selective electrochemical reduction of **1a** to **2a**.

As shown above, the diffraction peaks located at 31.774, 36.413, 46.282, 48.363, 56.489 and 76.081 of CoP after reaction could be indexed to the (011), (111), (112), (211), (301) and (222) plane oforthorhombic CoP (JCPDS No. 29-0497), highlighting its good stability in the crystalline structure of CoP after highly selective electrochemical reduction of **1a** to **2a** for six cycles.

**Supplementary Figure 10.** High resolution XPS spectra of CoP nanosheet cathode before and after electrochemical reduction of **1a** to **2a** for six cycles: a) Co 2p, b) P 2p.

The XPS, a surface-sensitive analytical technique, was adopted to study the surface chemical changes of the as-prepared CoP electrode before and after nitro-reduction reaction. Compared with the fresh CoP, the high-resolution Co 2p peaks of used CoP shifts to the higher binding energy slightly and the high-resolution P 2p XPS spectrum displays an intensity increase at 133.6 eV (P-O), suggesting the part oxidation of CoP. That might be ascribed to the exposure of CoP cathode to the air after the nitro-reduction reaction.

**Supplementary Figure 11.** Conversion and selectivity of paired electrochemical reduction and oxidation of **1a** to **2a** and octylamine to octylnitrile at CoP cathode and Ni_2_P anode, respectively, at -0.8 V vs. Ag/AgCl in 1.0 M KOH solution.

**Supplementary Figure 12.** Conversion and selectivity of electrochemical reduction of **1a** to **2a** over a CoP electrode at the potentials ranging from -0.65 to -0.95 V vs. Ag/AgCl in 1.0 M KOH solution.

**NMR and GC-MS data**

.

**Supplementary Figure 13. ^1^H NMR** (400 MHz, CDCl_3_) *δ* [ppm] 8.33 (d, *J* = 7.8 Hz, 2 H), 8.19 (d, *J* = 8.1 Hz, 2 H), 7.54 (ddd, *J* = 21.9, 14.4, 7.4 Hz, 5 H), 7.41 (t, *J* = 7.2 Hz, 1 H); **GC-MS** (EI) *m/z* 198.12, the theoretical value for C_12_H_10_N_2_O is 198.22.

**Supplementary Figure 14. ^13^C NMR** (101 MHz, CDCl_3_) *δ* [ppm] 148.36, 144.00, 131.62, 129.65, 128. 82, 128.74, 125.56,122.36.

**Supplementary Figure 15. ^1^H NMR** (400 MHz, CDCl_3_) *δ* [ppm] 8.23 (dd, *J* = 6.9, 5.0 Hz, 2 H), 8.20 – 8.13 (m, 2 H), 7.09 (dd, *J* = 14.5, 7.1 Hz, 4 H); **GC-MS** (EI) *m/z* 234.14, the theoretical value for C_12_H_8_F_2_N_2_O is 234.20.

**Supplementary Figure 16. ^13^C NMR** (101 MHz, CDCl_3_) *δ* [ppm] 164.81 (d, *J* = 197.0 Hz), 162.29 (d, *J* = 196.7 Hz), 140.29 (d, *J* = 2.8 Hz), 128.04 (d, *J* = 8.4 Hz), 124.30 (dd, *J* = 50.8, 7.5 Hz), 124.05 (d, *J* = 5.8 Hz), 115.84 (d, *J* = 5.5 Hz), 115.61 (d, *J* = 4.5 Hz).

**Supplementary Figure 17. ^1^H NMR** (400 MHz, CDCl_3_) *δ* [ppm] 8.24 (d, *J* = 8.1 Hz, 2 H), 8.16 (d, *J* = 8.1 Hz, 2 H), 7.52 – 7.38 (m, 4 H); **GC-MS** (EI) *m/z* 266.00, the theoretical value for C_12_H_8_Cl_2_N_2_O is 267.11.

**Supplementary Figure 18. ^13^C NMR** (101 MHz, CDCl_3_) *δ* [ppm] 146.53, 142.22, 138.09, 135.28, 129.04, 128.99, 127.10, 123.72.

**Supplementary Figure 19. ^1^H NMR** (400 MHz, CDCl_3_) *δ* [ppm] 8.17 (d, *J* = 8.5 Hz, 2 H), 8.11 (d, *J* = 8.4 Hz, 2 H), 7.26 (d, *J* = 8.0 Hz, 4 H), 2.41 (d, *J* = 8.5 Hz, 6 H); **GC-MS** (EI) *m/z* 226.10, the theoretical value for C_14_H_14_N_2_O is 226.27.

**Supplementary Figure 20. ^13^C NMR** (101 MHz, CDCl_3_) *δ* [ppm] 146.44, 142.0.09, 142.05, 140.18, 129.48, 125.84, 122.33, 21.73, 21.47.

**Supplementary Figure 21.** **^1^H NMR** (400 MHz, CDCl_3_) *δ* [ppm] 8.27 (dd, *J* = 14.1, 8.6 Hz, 4 H), 6.97 (t, *J* = 7.3 Hz, 4 H), 3.88 (s, 6 H); **GC-MS** (EI) *m/z* 258.14, the theoretical value for C_14_H_14_N_2_O_3_ is 258.27.

**Supplementary Figure 22. ^13^C NMR** (101 MHz, CDCl_3_) *δ* [ppm] 162.06, 160.40, 141.94, 138.22, 128.02, 123.99, 113.94, 113.81, 55.90, 55.71.

**Supplementary Figure 23. ^1^H NMR** (400 MHz, CDCl_3_) *δ* [ppm] 8.25 (dd, *J* = 17.2, 8.3 Hz, 4 H), 6.95 (t, *J* = 7.9 Hz, 4 H), 4.10 (q, *J* = 6.7 Hz, 4 H), 1.45 (t, *J* = 6.6 Hz, 6 H); **GC-MS** (EI) *m/z* 286.12, the theoretical value for C_16_H_18_N_2_O_3_ is 286.33.

**Supplementary Figure 24. ^13^C NMR** (101 MHz, CDCl_3_) *δ* [ppm] 161.43, 159.81, 141.73, 138.05, 128.02, 123.94, 114.38, 114.22, 64.16, 63.90, 14.97, 14.92.

**Supplementary Figure 25. ^1^H NMR** (400 MHz, CDCl_3_) *δ* [ppm] 8.07 – 7.87 (m, 3 H), 7.74 (d, *J* = 8.1 Hz, 1 H), 7.36 (dq, *J* = 16.0, 7.9 Hz, 2 H), 7.18 (t, *J* = 7.9 Hz, 1 H), 7.02 (t, *J* = 8.2 Hz, 1 H); **GC-MS** (EI) *m/z* 234.03, the theoretical value for C_12_H_8_F_2_N_2_O is 234.20.

**Supplementary Figure 26. ^13^C NMR** (101 MHz, CDCl_3_) *δ* [ppm] 163.64 (d, *J* = 7.7 Hz), 161.19 (d, *J* = 5.4 Hz), 149.33 (d, *J* = 8.6 Hz), 144.86 (d, *J* = 9.3 Hz), 130.16 (d, *J* = 8.4 Hz), 129.81 (d, *J* = 8.8 Hz), 122.21 (d, *J* = 2.6 Hz), 119.05 (d, *J* = 21.4 Hz), 118.16 (d, *J* = 3.2 Hz), 116.92 (d, *J* = 21.7 Hz), 112.27 (d, *J* = 24.7 Hz), 110.33 (d, *J* = 26.8 Hz).

**Supplementary Figure 27. ^1^H NM**R (400 MHz, CDCl_3_) *δ* [ppm] 8.31(s, 1 H), 8.27 (s, 1 H), 8.22 – 8.15 (m, 1 H), 7.99 (d, *J* = 7.6 Hz, 1 H), 7.54 (d, *J* = 7.8 Hz, 1 H), 7.48 – 7.34 (m, 3 H); **GC-MS** (EI) *m/z* 266.00, the theoretical value for C_12_H_8_Cl_2_N_2_O is 267.11.

**Supplementary Figure 28. ^13^C NMR** (101 MHz, CDCl_3_) *δ* [ppm] 148.96, 144.67, 134.98, 134.57, 132.19, 130.23 – 129.73 (m), 125.60, 124.28, 123.01, 120.78.

**Supplementary Figure 29. ^1^H NMR** (400 MHz, CDCl_3_) *δ* [ppm] 8.10 (d, *J* = 11.9 Hz, 2 H), 7.99 (d, *J* = 7.8 Hz, 2 H), 7.38 (d, *J* = 7.9 Hz, 3 H), 7.23 (t, *J* = 9.9 Hz, 1 H), 2.47 (s, 3 H), 2.44 (s, 3 H); **GC-MS** (EI) *m/z* 226.10, the theoretical value for C_14_H_14_N_2_O is 226.27.

**Supplementary Figure 30. ^13^C NMR** (101 MHz, CDCl_3_) *δ* [ppm] 148.46, 144.07, 139.02, 138.49, 132.35, 130.43, 128.64, 128.55, 126.08, 122.82, 122.57, 119.55, 21.54, 21.46.

**Supplementary Figure 31. ^1^H NMR** (400 MHz, CDCl_3_) *δ* [ppm] 8.36 (t, *J* = 7.7 Hz, 1 H), 7.99 (t, *J* = 7.6 Hz, 1 H), 7.58 (dd, *J* = 12.5, 7.6 Hz, 1 H), 7.44 (dd, *J* = 13.2, 7.3 Hz, 1 H), 7.39 – 7.27 (m, 4 H); **GC-MS** (EI) *m/z* 234.03, the theoretical value for C_12_H_8_F_2_N_2_O is 234.20.

**Supplementary Figure 32. ^13^C NMR** (101 MHz, CDCl_3_) *δ* [ppm] 156.88 (d, *J* = 168.2 Hz), 154.32 (d, *J* = 171.2 Hz), 132.49 (d, *J* = 8.0 Hz), 132.34 (d, *J* = 9.0 Hz), 130.97 (d, *J* = 3.7 Hz), 130.71 (d, *J* = 8.3 Hz), 128.85 (d, *J* = 1.5 Hz), 125.56 (d, *J* = 0.8 Hz), 124.42 (d, *J* = 4.1 Hz), 124.06 (d, *J* = 5.3 Hz), 117.76 (d, *J* = 20.2 Hz), 116.26 (d, *J* = 20.1 Hz).

**Supplementary Figure 33. ^1^H NMR** (400 MHz, CDCl_3_) *δ* [ppm] 8.01 (d, *J* = 8.0 Hz, 1 H), 7.76 (d, *J* = 7.3 Hz, 1 H), 7.54 (t, *J* = 8.2 Hz, 2 H), 7.49 – 7.35 (m, 3 H), 7.31 (t, *J* = 7.6 Hz, 1 H); **GC-MS** (EI) *m/z* 266.00, the theoretical value for C_12_H_8_Cl_2_N_2_O is 267.11.

**Supplementary Figure 34. ^13^C NMR** (101 MHz, CDCl_3_) *δ* [ppm] 147.48, 141.07, 131.36, 131.26, 130.48, 129.96, 129.77, 127.78, 127.24, 127.02, 125.39, 123.58.

**Supplementary Figure 35. ^1^H NMR** (400 MHz, CDCl_3_) *δ* [ppm] 8.17 (d, *J* = 8.3 Hz, 2 H), 8.08 (d, *J* = 8.2 Hz, 2 H), 7.62 (dd, *J* = 14.3, 8.4 Hz, 4 H); **GC-MS** (EI) *m/z* 356.10, the theoretical value for C_12_H_8_Br_2_N_2_O is 356.01.

**Supplementary Figure 36. ^13^C NMR** (101 MHz, CDCl_3_) *δ* [ppm] 147.21, 142.76, 132.23, 132.17, 127.44, 126.68, 124.09, 123.83.

**Supplementary Figure 37. ^1^H NMR** (400 MHz, CDCl_3_) *δ* [ppm] 8.24 (dd, *J* = 26.0, 7.9 Hz, 4 H), 7.53 (d, *J* = 7.3 Hz, 4 H), 6.89 – 6.68 (m, 2 H), 6.02 – 5.77 (m, 2 H), 5.57 – 5.28 (m, 2 H); **GC-MS** (EI) *m/z* 250.21, the theoretical value for C_16_H_14_N_2_O is 250.30.

**Supplementary Figure 38. ^13^C NMR** (101 MHz, CDCl_3_) *δ* [ppm] 147.67, 144.00, 143.70, 140.98, 139.05, 136.34, 135.72, 135.18, 127.02, 126.69, 126.29, 124.22, 122.74, 118.82,116.83, 116.02.

**Supplementary Figure 39. ^1^H NMR** (400 MHz, CDCl_3_) *δ* [ppm] 9.54 (s, 1 H), 9.21 (s, 1 H), 8.76 (dd, *J* = 25.5, 6.1 Hz, 2 H), 8.58 (dd, *J* = 12.9, 6.2 Hz, 2 H), 7.54 – 7.37 (m, 2 H); **GC-MS** (EI) *m/z* 199.99, the theoretical value for C_10_H_8_N_4_O is 200.20.

**Supplementary Figure 40. ^13^C NMR** (101 MHz, CDCl3) *δ* [ppm] 152.77, 150.59, 148.43, 144.22 (d, *J* = 18.6 Hz), 140.05, 131.62, 130.05, 123.63 (d, *J* = 7.0 Hz).

**Supplementary Figure 41. ^1^H NMR** (400 MHz, CDCl_3_) *δ* [ppm] 8.31 (dd, *J* = 22.3, 7.6 Hz, 6 H), 8.13 (d, *J* = 7.7 Hz, 2 H), 7.62 – 7.33 (m, 8 H), 6.98 (t, *J* = 8.7 Hz, 2 H), 3.89 (s, 6 H); **GC-MS** (EI) *m/z* 228.05, the theoretical value for C_13_H_12_N_2_O_2_ is 228.25

**Supplementary Figure 42. ^13^C NMR** (101 MHz, CDCl_3_) *δ* [ppm] 162.46, 160.71, 145.26, 144.41, 131.34, 129.42, 128.95, 128.87, 128.26, 125.57, 124.26, 122.36, 113.99, 113.85, 55.93, 55.72.

**Supplementary Figure 43. ^1^H NMR** (400 MHz, CDCl_3_) *δ* [ppm] 8.38 – 8.17 (m, 8 H), 7.15 (t, *J* = 8.2 Hz, 4 H), 6.97 (d, *J* = 8.4 Hz, 4 H), 3.89 (s, 6 H); **GC-MS** (EI) *m/z* 246.03, the theoretical value for C_13_H_11_FN_2_O_2_ is 246.24.

**Supplementary Figure 44. ^13^C NMR** (101 MHz, CDCl_3_) *δ* [ppm] 163.17, 162.42, 161.19,160.79, 140.74, 137.91, 131.13, 129.04, 128.24, 127.97 (d, *J* = 8.3 Hz), 124.54 (d, *J* = 9.1 Hz), 124.16, 115.77 (dd, *J* = 22.9, 4.3 Hz), 113.94 (d, *J* = 14.2 Hz), 55.92, 55.72.

**Supplementary Figure 45. (1): ^1^H NMR** (400 MHz, CDCl_3_) *δ* [ppm] 8.32 (d, *J* = 8.3 Hz, 2 H), 8.25 (d, *J* = 8.0 Hz, 2 H), 7.46 (d, *J* = 8.1 Hz, 2 H), 6.99 (d, *J* = 8.3 Hz, 2 H), 3.88 (s, 3 H); **GC-MS** (EI) *m/z* 261.99, the theoretical value for C_13_H_11_ClN_2_O_2_ is 262.69.

**Supplementary Figure 46. (1) ^13^C NMR** (101 MHz, CDCl_3_) *δ* [ppm] 160.86, 146.83, 137.83, 137.44, 129.01, 128.28, 123.6, 113.97, 55.66.

**Supplementary Figure 47. (2)** **^1^H NMR** (400 MHz, CDCl_3_) *δ* [ppm] 8.26 (d, *J* = 7.9 Hz, 2 H), 8.12 (d, *J* = 7.6 Hz, 2 H), 7.43 (d, *J* = 7.6 Hz, 2 H), 6.97 (d, *J* = 8.0 Hz, 2 H), 3.89 (s, 3 H); **GC-MS** (EI) *m/z* 261.99, the theoretical value for C_13_H_11_ClN_2_O_2_ is 262.69.

**Supplementary Figure 48. (2) ^13^C NMR** (101 MHz, CDCl_3_) *δ* [ppm] 162.52, 142.76, 141.65, 134.60, 129.00, 126.99, 124.20, 113.86, 55.90.

**Supplementary Figure 49. ^1^H NMR** (400 MHz, CDCl_3_) *δ* [ppm] 8.42 – 8.19 (m, 4 H), 8.15 – 7.97 (m, 3 H), 7.79 (d, *J* = 7.0 Hz, 1 H), 7.46 (ddd, *J* = 18.2, 16.5, 12.3 Hz, 3 H), 7.13 – 6.93 (m, 5 H), 3.90 (s, 6 H); **GC-MS** (EI) *m/z* 246.04, the theoretical value for C_13_H_11_FN_2_O_2_ is 246.24.

**Supplementary Figure 50. ^13^C NMR** (101 MHz, CDCl_3_) *δ* [ppm] 163.87 (d, *J* = 5.2 Hz), 162.6, 161.41 (d, *J* = 3.0 Hz), 161.04, 145.57 (d, *J* = 9.0 Hz), 137.82, 130.15 (d, *J* = 8.5 Hz), 129.85 (d, *J* = 8.8 Hz), 129.05, 128.46, 124.32, 121.96 (d, *J* = 2.9 Hz), 118.50 , 118.39 – 117.94 (m), 116.32, 116.10, 113.99 (d, *J* = 15.0 Hz), 112.32, 112.08, 110.41, 110.14, 55.95, 55.75.

**Supplementary Figure 51. ^1^H NMR** (400 MHz, CDCl_3_) *δ* [ppm] 8.33 (d, *J* = 8.7 Hz, 2 H), 7.89 (t, *J* = 7.6 Hz, 1 H), 7.49 (s, 1 H), 7.30 (d, *J* = 9.2 Hz, 2 H), 7.01 (d, *J* = 8.7 Hz, 2 H), 3.91 (s, 3 H); **GC-MS** (EI) *m/z* 246.03, the theoretical value for C_13_H_11_FN_2_O_2_ is 246.24.

**Supplementary Figure 52. ^13^C NMR** (101 MHz, CDCl_3_) *δ* [ppm] 160.89, 154.6 (d, *J* = 256.0 Hz), 137.96, 131.66 (d, *J* = 7.8 Hz), 128.09, 125.36, 124.32 (d, *J* = 3.5 Hz), 117.68, 117.48, 114.03, 113.78, 77.20 (d, *J* = 32.0 Hz), 55.56.

**Supplementary Figure 53. ^1^H NMR** (400 MHz, CDCl_3_) *δ* [ppm] 8.39 – 8.20 (m, 3 H), 8.12 (d, *J* = 8.2 Hz, 1 H), 7.53 – 7.34 (m, 3 H), 6.96 (t, *J* = 8.4 Hz, 2 H), 4.11 (q, *J* = 6.9 Hz, 2 H), 1.50 – 1.41 (m, 3 H); **GC-MS** (EI) *m/z* 242.10, the theoretical value for C_14_H_14_N_2_O_2_ is 242.27.

**Supplementary Figure 54. ^13^C NMR** (101 MHz, CDCl_3_) *δ* [ppm] 161.84, 160.16, 144.36, 137.93, 131.30, 129.39, 128.95, 128.87, 128.29, 125.55, 124.24, 122.34, 114.43, 114.27, 64.22, 63.95, 14.96, 14.91.

**Supplementary Figure 55. ^1^H NMR** (400 MHz, CDCl_3_) *δ* [ppm] 2.26 (t, *J* = 7.1 Hz, 2 H), 1.66 – 1.50 (m, 2 H), 1.37 (dd, *J* = 14.5, 6.9 Hz, 2 H), 1.24 (d, *J* = 8.4 Hz, 6 H), 0.82 (t, *J* = 6.1 Hz, 3 H); **GC-MS** (EI) *m/z* 109.99, the theoretical value for C_8_H_15_N is 110.08.

**Supplementary Figure 56. ^13^C NMR** (101 MHz, CDCl_3_) *δ* [ppm] 119.96, 31.62, 28.76, 28.56, 25.52, 22.64, 17.24, 14.12.

**Supplementary Figure 57. ^1^H NMR** (400 MHz, CDCl_3_) *δ* [ppm] 7.96 (d, *J* = 7.4 Hz, 4 H), 7.57 – 7.47 (m, 6 H); **GC-MS** (EI) *m/z* 182.02, the theoretical value for C_12_H_10_N_2_ is 182.22.

**Supplementary Figure 58. ^13^C NMR** (101 MHz, CDCl_3_) *δ* [ppm] 152.89, 131.18, 129.29, 123.06.

**Supplementary Figure 59. ^1^H NMR** (400 MHz, CDCl_3_) *δ* [ppm] 7.81 (d, *J* = 7.7 Hz, 4 H), 7.31 (d, *J* = 7.6 Hz, 4 H), 2.43 (s, 6 H); **GC-MS** (EI) *m/z* 210.1, the theoretical value for C_14_H_14_N_2_ is 210.27.

**Supplementary Figure 60. ^13^C NMR** (101 MHz, CDCl_3_) *δ* [ppm] 151.07, 141.41, 129.92, 122.94 , 21.68.

**Supplementary Figure 61. ^1^H NMR** (400 MHz, CDCl_3_) *δ* [ppm] 7.86 (d, *J* = 8.6 Hz, 4 H), 7.49 (d, *J* = 8.6 Hz, 4 H); **GC-MS** (EI) *m/z* 251.01, the theoretical value for C_12_H_8_Cl_2_N_2_ is 251.11

**Supplementary Figure 62. ^13^CNMR** (101 MHz, CDCl_3_) *δ* [ppm] 151.09, 137.48, 129.64, 124.42.

**Supplementary Figure 63. ^1^H NMR** (400 MHz, CDCl_3_) *δ* [ppm] 7.77 (d, *J* = 7.8 Hz, 2 H), 7.61 (d, *J* = 9.7 Hz, 2 H), 7.51 (dd, *J* = 14.3, 7.2 Hz, 2 H), 7.21 (t, *J* = 8.1 Hz, 2 H); **GC-MS** (EI) *m/z* 218.03, the theoretical value for C_12_H_8_F_2_N_2_ is 218.20.

**Supplementary Figure 64. ^13^C NMR** (101 MHz, CDCl_3_) *δ* [ppm] 163.5 (d, *J* =247.0 Hz), 154.03 (d, *J* = 7.5 Hz), 130.56 (d, *J* = 8.6 Hz), 120.99 (d, *J* = 2.8 Hz), 118.53, 118.31, 108.50, 108.27.

**Supplementary Figure 65. ^1^H NMR** (400 MHz, CDCl_3_) *δ* [ppm] 7.72 (s, 4 H), 7.41 (t, *J* = 7.2 Hz, 2 H), 7.29 (d, *J* = 7.2 Hz, 2 H), 2.46 (s, 6 H); **GC-MS** (EI) *m/z* 218.03, the theoretical value for C_14_H_14_N_2_ is 218.27.

**Supplementary Figure 66. ^13^C NMR** (101 MHz, CDCl_3_) *δ* [ppm] 153.05, 139.19, 131.89, 129.11, 123.09, 120.67, 21.57.

**Supplementary Figure 67. ^1^H NMR** (400 MHz, CDCl_3_) *δ* [ppm] 9.18 (s, 2 H), 8.69 (d, *J* = 2.0 Hz, 2 H), 8.12 (d, *J* = 8.1 Hz, 2 H), 7.44 – 7.39 (m, 2 H); **GC-MS** (EI) *m/z* 183.81, the theoretical value for C_10_H_8_N_4_ is 184.20.

**Supplementary Figure 68. ^13^C NMR** (101 MHz, CDCl_3_) *δ* [ppm] 152.51, 150.59，147.78, 126.97, 124.15.

**Supplementary Figure 69. ^1^H NMR** (400 MHz, CDCl_3_) *δ* [ppm] 7.12 (s, 2 H), 6.73 (d, *J* = 3.5 Hz, 1 H), 6.68 – 6.53 (m, 2 H), 3.55 (s, 2 H); **GC-MS** (EI) *m/z* 93.06, the theoretical value for C_12_H_10_N_2_O is 93.14.

**Supplementary Figure 70. ^13^C NMR** (101 MHz, CDCl_3_) *δ*[ppm] 146.50, 129.57, 129.29, 118.44, 115.37, 115.09.

**Supplementary Figure 71. ^1^H NMR** (400 MHz, CDCl_3_) *δ* [ppm] 6.99 (d, *J* = 7.3 Hz, 2 H), 6.63 (d, *J* = 7.1 Hz, 2 H), 3.52 (s, 2 H), 2.27 (s, 3 H); **GC-MS** (EI) *m/z* 106.98, the theoretical value for C_7_H_9_N is 107.15.

**Supplementary Figure 72. ^13^C NMR** (101 MHz, CDCl_3_) *δ* [ppm] 143.95, 129.91, 127.93, 115.43, 20.61.

**Supplementary Figure 73. ^1^H NMR** (400 MHz, CDCl_3_) *δ* [ppm] (d, *J* = 8.5 Hz, 2 H), 6.64 (d, *J* = 8.3 Hz, 2 H), 3.75 (s, 3 H), 3.47 (s, 2 H); **GC-MS** (EI) *m/z* 123.22, the theoretical value for C_7_H_9_NO is 123.16.

**Supplementary Figure 74. ^13^C NMR** (101 MHz, CDCl_3_) *δ* [ppm]152.69, 140.12, 116.39, 114.80, 55.70.

**Supplementary Figure 75. ^1^H NMR** (400 MHz, CDCl_3_) *δ* [ppm] 6.74 (d, *J* = 8.3 Hz, 2 H), 6.64 (d, *J* = 8.3 Hz, 2 H), 3.95 (q, *J* = 6.9 Hz, 2 H), 3.35 (s, 2 H), 1.37 (t, *J* = 6.9 Hz, 3 H); **GC-MS** (EI) *m/z* 136.96, the theoretical value for C_8_H_11_NO is 137.18.

**Supplementary Figure 76. ^13^C NMR** (101 MHz, CDCl_3_) *δ* [ppm] 152.28, 140.06, 116.60, 115.83, 64.21, 15.18.

**Supplementary Figure 77. ^1^H NMR** (400 MHz, CDCl_3_) *δ* [ppm] 6.57 (s, 4 H), 3.29 (s, 4 H); **GC-MS** (EI) *m/z* 108.04, the theoretical value for C_6_H_8_N_2_ is 108.14.

**Supplementary Figure 78. ^13^C NMR** (101 MHz, CDCl_3_) *δ* [ppm] 138.74, 116.88.

**Supplementary Figure 79. ^1^H NMR** (400 MHz, DMSO) *δ* [ppm] 8.38 (s, 1 H), 6.47 (dd, *J* = 25.7, 8.3 Hz, 4 H), 4.36 (s, 2 H); **GC-MS** (EI) *m/z* 119.04, the theoretical value for C_6_H_7_NO is 109.13.

**Supplementary Figure 80.** **^13^C NMR** (101 MHz, DMSO) *δ* [ppm] 148.33, 140.64, 115.64, 115.38.

**Supplementary Figure 81. ^1^H NMR** (400 MHz, CDCl_3_) *δ* [ppm] 6.87 (t, *J* = 8.4 Hz, 2 H), 6.65 – 6.56 (m, 2 H), 3.56 (s, 2 H); **GC-MS** (EI) *m/z* 111.01, the theoretical value for C_6_H_6_FN is 111.12.

**Supplementary Figure 82. ^13^C NMR** (101 MHz, CDCl_3_) *δ* [ppm] 157.64, 155.30, 142.59, 116.16 (d, *J* = 7.6 Hz), 115.85, 115.63.

**Supplementary Figure 83. ^1^H NMR** (400 MHz, CDCl_3_) *δ* [ppm] 7.10 (d, *J* = 8.6 Hz, 2 H), 6.60 (d, *J* = 8.6 Hz, 2 H), 3.66 (s, 2 H); **GC-MS** (EI) *m/z* 126.88, the theoretical value for C_6_H_6_ClN is 127.57.

**Supplementary Figure 84. ^13^C NMR** (101 MHz, CDCl_3_) *δ* [ppm] 145.12, 129.26, 123.26, 116.39.

**Supplementary Figure 85. ^1^H NMR** (400 MHz, CDCl_3_) *δ* [ppm] 7.07 (t, *J* = 7.4 Hz, 1 H), 6.61 (d, *J* = 7.2 Hz, 1 H), 6.49 (d, *J* = 7.5 Hz, 2 H), 3.58 (s, 2 H), 2.29 (s, 3 H); **GC-MS** (EI) *m/z* 106.98, the theoretical value for C_7_H_9_N is 107.15.

**Supplementary Figure 86. ^13^C NMR** (101 MHz, CDCl_3_) *δ* [ppm] 146.49, 139.13, 129.22, 119.44, 115.97, 112.31, 21.50.

**Supplementary Figure 87. ^1^H NMR** (400 MHz, CDCl_3_) *δ* [ppm] 7.12 (dd, *J* = 15.3, 7.5 Hz, 1 H), 6.54 – 6.36 (m, 3 H), 3.81 (s, 2 H); **GC-MS** (EI) *m/z* 111.01, the theoretical value for C_6_H_6_FN is 111.11.

**Supplementary Figure 88. ^13^C NMR** (101 MHz, CDCl_3_) *δ* [ppm] 165.09, 162.68, 148.47 (d, *J* = 10.8 Hz), 130.50 (d, *J* = 10.0 Hz), 110.78 (d, *J* = 2.3 Hz), 104.99, 104.78, 102.09, 101.84.

**Supplementary Figure 89. ^1^H NMR** (400 MHz, CDCl_3_) *δ* [ppm] 7.08 (t, *J* = 8.0 Hz, 1 H), 6.76 (dd, *J* = 7.9, 0.8 Hz, 1 H), 6.67 (d, *J* = 1.3 Hz, 1 H), 6.54 (d, *J* = 8.1 Hz, 1 H), 3.74 (s, 2 H); **GC-MS** (EI) *m/z* 126.85, the theoretical value for C_6_H_6_ClN is 127.57.

**Supplementary Figure 90. ^13^C NMR** (101 MHz, CDCl_3_) *δ* [ppm] 147.79 , 134.77, 130.40, 118.38, 114.92 (s), 113.29.

**Supplementary Figure 91. ^1^H NMR** (400 MHz, CDCl_3_) *δ* [ppm] 7.14 (d, *J* = 7.4 Hz, 2 H), 6.85 (t, *J* = 7.4 Hz, 1 H), 3.68 (s, 2 H), 2.35 (s, 6 H); **GC-MS** (EI) *m/z* 121.08, the theoretical value for C_8_H_11_N is 121.18.

**Supplementary Figure 92. ^13^C NMR** (101 MHz, CDCl_3_) *δ* [ppm] 142.79, 128.11 (d, *J* = 29.5 Hz), 121.61, 117.94, 17.55.

**Supplementary Figure 93. ^1^H NMR** (400 MHz, CDCl_3_) *δ* [ppm] 6.75 (dd, *J* = 17.6, 9.1 Hz, 1 H), 6.49 – 6.38 (m, 1 H), 3.68 (s, 2 H); **GC-MS** (EI) *m/z* 147.00, the theoretical value for C_6_H_4_F_3_N is 147.09.

**Supplementary Figure 94. ^13^C NMR** (101 MHz, CDCl_3_) *δ* [ppm] 145.33 (d, *J* = 10.0 Hz), 142.95 (d, *J* = 10.8 Hz), 142.28 – 141.51 (m), 139.47 (ddd, *J* = 29.7, 14.1, 8.3 Hz), 111.48 (dd, *J* = 18.0, 3.9 Hz), 109.77 (dt, *J* = 7.1, 3.6 Hz).

**Supplementary Figure 95. ^1^H NMR** (400 MHz, CDCl_3_) *δ* [ppm] 6.73 (d, *J* = 3.8 Hz, 4 H), 3.38 (s, 4 H); **GC-MS** (EI) *m/z* 108.04, the theoretical value for C_6_H_7_NO is 108.13

**Supplementary Figure 96. ^13^C NMR** (101 MHz, CDCl_3_) *δ* [ppm] 134.88, 120.39, 116.88.

**Supplementary Figure 97. ^1^H NMR** (400 MHz, DMSO) *δ* [ppm] 7.94 (s, 1 H), 7.73 (s, 1 H), 7.04 – 6.96 (m, 1 H), 6.91 (d, *J* = 7.4 Hz, 1 H), 5.25 (s, 2 H); **GC-MS** (EI) *m/z* 94.05, the theoretical value for C_5_H_6_N_2_ is 94.12.

**Supplementary Figure 98. ^13^C NMR** (101 MHz, DMSO) *δ* [ppm] 144.80, 136.93, 136.39, 123.55, 119.66.

**Supplementary Figure 99. ^1^H NMR** (400 MHz, CDCl_3_) *δ* [ppm] 6.93 – 6.64 (m, 4 H), 3.89 (s, 3 H), 3.83 (s, 2 H). **GC-MS** (EI) *m/z* 122.93, the theoretical value for C_7_H_9_NO is 123.15 .

**Supplementary Figure 100. ^13^C NMR** (101 MHz, CDCl_3_) *δ* [ppm] 147.35, 136.29, 121.13, 118.43, 115.04, 110.51, 55.43.

**Supplementary Figure 101. ^1^H NMR** (400 MHz, CDCl_3_) *δ* [ppm] 6.73 (d, *J* = 3.5 Hz, 4 H), 3.38 (s, 4 H); **GC-MS** (EI) *m/z* 108.04, the theoretical value for C_6_H_8_N_2_ is 108.14.

**Supplementary Figure 102. ^13^C NMR** (101 MHz, CDCl_3_) *δ* [ppm] 134.88, 120.39, 116.87 .

**Supplementary Figure 103. ^1^H NMR** (400 MHz, DMSO) *δ* [ppm] 8.93 (s, 1 H), 6.70 – 6.36 (m, 4 H), 4.45 (s, 2 H); **GC-MS** (EI) *m/z* 109.02, the theoretical value for C_6_H_7_NO is 109.13.

**Supplementary Figure 104. ^13^C NMR** (101 MHz, DMSO) *δ* [ppm] 144.04, 136.52, 119.57, 116.53, 114.49 (d, *J* = 8.7 Hz).

**Supplementary Figure 105. ^1^H NMR** (400 MHz, CDCl_3_) *δ* [ppm] 7.01 (dt, *J* = 15.6, 8.3 Hz, 2 H), 6.87 – 6.61 (m, 2 H), 3.76 (s, 2 H); **GC-MS** (EI) *m/z* 111.01, the theoretical value for C_6_H_6_FN is 111.11.

**Supplementary Figure 106. ^13^C NMR** (101 MHz, CDCl_3_) *δ* [ppm] 152.99, 150.62, 134.64 (d, *J* = 12.8 Hz), 124.55 (d, *J* = 3.5 Hz), 118.67 (d, *J* = 6.8 Hz), 117.05 (d, *J* = 3.5 Hz), 115.29 (d, *J* = 18.4 Hz).

**Supplementary Figure 107. ^1^H NMR** (400 MHz, CDCl_3_) *δ* [ppm] 7.00 (t, *J* = 7.9 Hz, 1 H), 6.91 – 6.78 (m, 2 H), 6.58 (d, *J* = 7.9 Hz, 1 H), 3.64 (s, 2 H); **GC-MS** (EI) *m/z* 172.13, the theoretical value for C_6_H_6_BrN is 172.02.

**Supplementary Figure 108.** **^13^C NMR** (101 MHz, CDCl_3_) *δ* [ppm] 147.97, 130.77, 123.19, 121.50, 117.97, 113.79.

**Supplementary Figure 109. ^1^H NMR** (400 MHz, CDCl_3_) *δ* [ppm] 7.77 (d, *J* = 5.3 Hz, 2 H), 6.62 (d, *J* = 5.7 Hz, 2 H), 4.28 (s, 2 H), 2.48 (s, 3 H); **GC-MS** (EI) *m/z* 134.95, the theoretical value for C_8_H_9_NO is 135.17.

**.**

**Supplementary Figure 110. ^13^C NMR** (101 MHz, CDCl_3_) *δ* [ppm] 196.70, 151.55, 130.90, 127.70, 113.78 , 26.15.

**Supplementary Figure 111.  ^1^H NMR** (400 MHz, CDCl_3_) *δ* [ppm] 7.22 (d, *J* = 8.1 Hz, 2 H), 6.63 (d, *J* = 8.2 Hz, 3 H), 5.54 (d, *J* = 17.6 Hz, 1 H), 5.04 (d, *J* = 10.9 Hz, 1 H), 3.57 (s, 2 H); **GC-MS** (EI) *m/z* 119.04, the theoretical value for C_8_H_9_N is 119.16.

**Supplementary Figure 112. ^13^C NMR** (101 MHz, CDCl_3_) *δ* [ppm] 146.35, 136.71, 128.53, 127.53, 115.19, 110.19.

**Supplementary Figure 113.** **^1^H NMR** (400 MHz, CDCl_3_) *δ* [ppm] 7.19 (t, *J* = 7.5 Hz, 2 H), 6.82 (t, *J* = 7.1 Hz, 3 H); **GC-MS** (EI) *m/z* 94.86, the theoretical value for C_8_D_9_N is 95.15; **HRMS** (EI) *m/z* 95.0687, the theoretical value for C_8_D_9_N is 95.0704.

**.**

**Supplementary Figure 114.** ^13^C NMR (101 MHz, CDCl_3_) *δ* [ppm] 149.03, 129.52, 120.04, 112.50.

**References**

[1] C. Zhang, Y. Huang, Y. Yu, J. Zhang, S. Zhuo, B. Zhang, *Chem. Sci.* **2017**, *8*, 2769-2775.

[2] J. Wang, W. Cui , Q. Liu , Z. Xing , A. M. Asiri, X. Sun, *Adv. Mater.* **2016**, *28*, 215-230.

[3] Z. H. Xue, H. Su, Q. Y. Yu, B. Zhang, H. H. Wang, X. H. Li, J. S. Chen, *Adv. Energy Mater.* **2017**, *7*, 1602355.

[4] N. Jiang, B. You, M. Sheng, Y. Sun, *Angew. Chem. Int. Ed.* **2015**, *54*, 6251-6254; *Angew. Chem.* **2015**, *127*, 6349-6352.
